# Electroencephalographic markers in Major Depressive Disorder: insights from absolute, relative power, and asymmetry analyses

**DOI:** 10.3389/fpsyt.2024.1480228

**Published:** 2025-01-13

**Authors:** Mehmet Akif Özçoban, Oğuz Tan

**Affiliations:** ^1^ Electronic and Automation Department, Naci Topcuoglu Vocational School, Gaziantep University, Gaziantep, Türkiye; ^2^ Feneryolu Medical Center, Üsküdar University, Istanbul, Türkiye

**Keywords:** Major Depressive Disorders, EEG, asymmetry, absolute power, relative power

## Abstract

**Introduction:**

Major Depressive Disorder (MDD) leads to dysfunction and impairment in neurological structures and cognitive functions. Despite extensive research, the pathophysiological mechanisms and effects of MDD on the brain remain unclear. This study aims to assess the impact of MDD on brain activity using EEG power spectral analysis and asymmetry metrics.

**Methods:**

EEG recordings were obtained from 48 patients with MDD and 78 healthy controls. The data were segmented into 2-second windows (1024 data points) and analyzed using the Welch method, an advanced variant of the Fast Fourier Transform (FFT). A Hanning time window with 50% overlap was applied to compute the modified periodogram. Absolute and relative power, along with asymmetry values in the theta, alpha, and beta frequency bands, were calculated.

**Results:**

Patients with MDD exhibited significantly higher absolute and relative power in the theta and beta bands and decreased power in the alpha band compared to healthy controls. Asymmetry analysis revealed significant differences between symmetric channels in the theta band (F7-F8, C3-C4, T3-T4, T5-T6), alpha band (F7-F8, C3-C4, T3-T4, T5-T6, O1-O2), and beta band (C3-C4, T3-T4, T5-T6, P3-P4).

**Discussion:**

The findings suggest that MDD affects brain mechanisms and cognitive functions, as evidenced by altered power values in the theta and alpha bands. Additionally, asymmetry values in theta, alpha, and beta bands may serve as potential biomarkers for MDD. This study highlights that beyond the commonly used alpha asymmetry, theta and beta asymmetry can also provide valuable insights into the neurophysiological effects of MDD, aligning with previous neuroimaging studies that indicate impairments in memory, attention, and neuroanatomical connectivity in MDD.

## 1 Introduction

Major Depressive Disorder (MDD) is a neuropsychiatric condition that adversely affects mental health and is characterized by symptoms such as depressed mood, insomnia, loss of concentration, and feelings of worthlessness (1). It is the leading cause of illness-induced disability worldwide (2). MDD affects brain function and is associated with structural and functional abnormalities, as well as cognitive decline. Neuroimaging studies have shown that MDD induces neuropathological changes in various brain regions, with the hippocampus being the most extensively studied. Notably, many studies indicate that MDD is linked to hippocampal volume reduction (3–5). Similarly, volumetric reductions have been detected in the frontal regions, particularly in the orbitofrontal cortex (OFC) (6–8). The amygdala, a critical structure in emotional processing and behavior, has also been differentially examined in its left and right hemispheres (3, 4, 9). Changes in amygdala volume have been reported to vary depending on the severity of illness (5, 9, 10) and the patient’s gender (5, 11, 12). MDD also impacts the basal ganglia, which includes structures such as the caudate nucleus and globus pallidus, impairing their function (3, 13). Additionally, abnormalities in the dorsolateral prefrontal cortex (DLPFC) and anterior cingulate cortex (ACC) pathways have been observed (14). These neuroanatomical alterations contribute to the cognitive deficits experienced by patients with MDD (15). For instance, hippocampal volume reduction is closely associated with memory impairments (16–18). Furthermore, structural dysfunctions in the anterior caudate nucleus, hippocampal gyrus, insula, and cingulate cortex—regions involved in learning and emotional regulation—have been demonstrated (19, 20). Early diagnosis of MDD is crucial to prevent neural and cognitive impairments. Currently, diagnosis and symptom severity assessments are primarily based on questionnaires and clinical interviews, underscoring the need for objective diagnostic criteria in neuropsychiatric diseases. Bioelectrical signals provide valuable insights into the mental, cognitive, and functional state of the brain. Electroencephalography (EEG) is a cost-effective, accessible, and portable tool that offers critical information about brain physiology, neural activities, and the diagnosis of neuropsychiatric disorders. Due to these advantages, electro-neurophysiological biomarkers derived from EEG recordings are particularly suitable for developing objective diagnostic criteria and understanding the central nervous system’s biology in neuropsychiatric diseases (21). Clinical EEG studies have long contributed to the understanding of neuropsychiatric and neurological disorders, offering important insights into their dysfunctional effects. Several studies have identified EEG biomarkers associated with anxiety disorders, revealing reduced power in the prefrontal cortex (22). Absolute and relative power, as well as asymmetry values across different frequency bands, provide crucial information about the brain’s regional mechanisms underlying mood disorders. Elevated absolute (23–25) and relative alpha power (26) have been reported, especially in the parietal, frontal, and occipital regions (25, 27, 28) though some studies have not found significant differences in the alpha band (29, 30). Additionally, increased beta power has been observed in some cases (21). Interhemispheric asymmetry values, derived from power measurements, yield important information about brain activity and physiology.

The majority of studies on MDD focus on frontal alpha asymmetry, with some associating these values with neural systems and others interpreting them as diagnostic biomarkers. Resting-state EEG analysis suggests that individuals with MDD exhibit relatively increased right frontal activity (31) which correlates with symptom severity (32). Even in remitted MDD patients, reduced left frontal activity has been reported, indicating that these patterns can serve as biomarkers for MDD (33). Davidson et al. associated frontal alpha asymmetry with approach-avoidance mechanism (34).

The aim of this study was to explore the effects of MDD on brain wave activity across various frequency bands. Resting-state EEG recordings were obtained from both patients with MDD and healthy volunteers, and their results were compared. Statistical analyses were conducted to evaluate EEG parameters between the groups, aiming to uncover potential associations. We hypothesized that patients with MDD would demonstrate inattention symptoms, which would be reflected in qEEG changes, particularly in beta and low gamma bands. Therefore, the objectives of this study were to clarify whether inattention symptoms could be represented by qEEG band power and to explore the influence of anxiety and depressive symptom severity on inattention and qEEG power in patients with MDD.

## 2 Materials and methods

### 2.1 Data acquisition

EEG data were collected from age-matched healthy individuals and patients at the Üsküdar University Neuropsychiatry Health Practice and Research Center. Patients diagnosed with Major Depressive Disorder (MDD) were assessed using the Structured Clinical Interview for DSM-5 (SCID-5) at the Uskudar University Outpatient Clinic in Istanbul, Turkey. All patients were medication-free for at least two weeks prior to EEG acquisition. Each participant provided informed consent, and the study was conducted in accordance with the principles of the Declaration of Helsinki. The research protocol received approval from the university’s ethics committee.

The healthy control group consisted of individuals with no history of stroke, neurological, or neuropsychiatric disorders, and they were not taking any medication. Additionally, patients had never received psychiatric therapy at the clinic center. The EEG data group included 48 MDD patients (24 males and 24 females) and 78 healthy volunteers (39 males and 39 females). Additional demographic information is presented in **Table 1**.

**Table 1:** The demographic information and clinical characteristics of the patient and healthy groups.

	Patients	Healthy Control
**Participants**	48	78
**Age (mean ± SD)**	31 ± 9 years	32 ± 10 years
**Gender (M/F)**	24/24	39/39
**BDI (mean ± SD)**	22.5 ± 8	–
**BAI (mean ± SD)**	16 ± 8	–

The Beck Depression Inventory (BDI) and Beck Anxiety Inventory (BAI) are 21-item questionnaires designed from clinical observations of attitudes and symptoms commonly seen in depressed patients. The BDI is used to assess the severity of depressive symptoms, whereas the BAI evaluates anxiety symptom severity (35, 36).

EEG data were recorded over 7 minutes at a sampling rate of 512 Hz in a resting-state condition with participants’ eyes closed. Nineteen electrodes (Fp1, Fp2, F3, F4, F7, F8, C3, C4, T3, T4, P3, P4, T5, T6, O1, O2, Fz, Cz, and Pz) were positioned on the scalp based on the international 10-20 electrode placement system. The impedance for all electrodes was maintained below 5 kΩ. Artifacts caused by muscle movements or eye blinks, including electrooculogram (EOG) signals, were removed by a neurophysiology expert, with an amplitude threshold of 50 µV peak-to-peak. A band-pass filter (0.5-70 Hz) and a notch filter (50 Hz) were applied to the data using Scan Edit 4.3 software.

Spectral analyses and asymmetry calculations were conducted using MATLAB. Data underwent further filtering with a second-order high-pass zero-phase Butterworth filter at a cutoff frequency of 0.5 Hz to eliminate unwanted signals and direct current (DC) components. Additionally, a notch filter was applied to eliminate 50-Hz power-line interference. Independent Component Analysis (ICA) was also utilized to process and remove any remaining artifacts.

### 2.2 Welch method

A crucial method in signal processing, power spectrum estimation (PSE) shows how an energy of signal is distributed among its many frequency components. By using a smoothing function to lessen unpredictability, the Welch method—an enhanced version of the traditional periodogram—improves the estimation of power spectral density (37).

**Figure 1:**
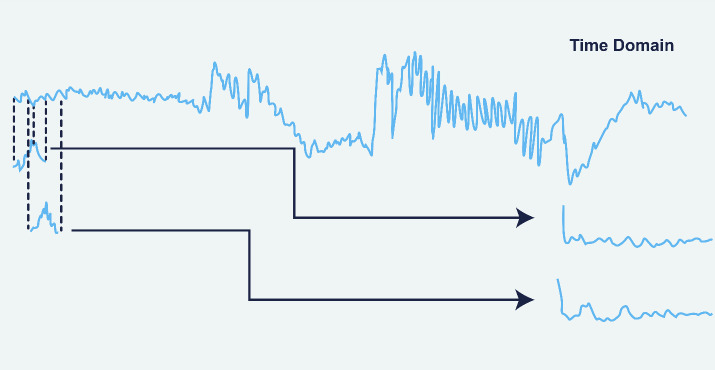
Application of the Welch Method on the EEG data (38).

The method is applied in several steps. First, the time-series signal was obtained from EEG data, is divided into overlapping parts of equal length, with a 50% overlap to minimize edge effects that could distort the spectrum. Each part, known as a window, is examined independently. In **Figure 1**, application of the Welch method is modelled (38).

**Figure 2:**
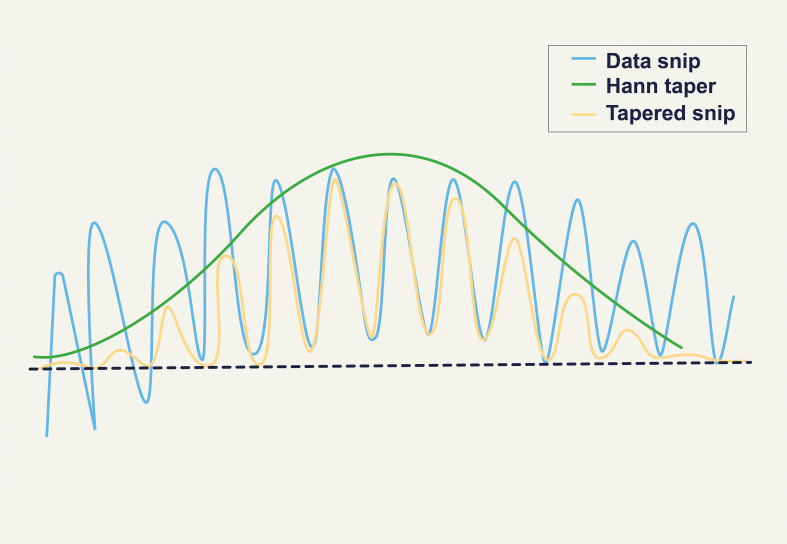
Effect of the Hanning Window (38).


Artifact-free continuous EEG data were split into 2-second epochs (1024 data points per window) for this investigation. Each segment’s power spectrum was calculated using a Hanning window, which trims each segment’s edges to minimize distortions. By lowering noise and spectrum leakage, this overlap and tapering increase the results’ dependability. The Hanning window is applied to obtain the modified periodogram of each segment of the signal. In **Figure 2**, the effect of the Hanning window is illustrated (38). In the Welch method, *L* represents the number of data segments of a given length. The parameter *M* is calculated based on these *L* data segments, and *U* is a normalization factor used to adjust the periodogram (37).

(1) $$U=\;\frac{1}{M}\sum_{n=0}^{M-1}w^{2}\left(n\right)$$

Welch power spectral density *P_WE_ (f):*


(2) $$P_{WE}=\frac{1}{L}\sum_{0}^{L-1}P_{j}\left(f\right)$$

In this method, a sliding window with a predefined length and overlap is applied to the signal, dividing it into successive time blocks, with a periodogram created for each block. In the Welch method, power spectral density is computed by first dividing the time-series data into segments. A periodogram is then constructed for each segment, and finally, these periodograms are averaged over time to estimate the overall power spectral density.

### 2.3 Inter-hemispheric asymmetry

Inter hemispheric asymmetry scores were computed separately for each EEG channel and frequency band. Inıtially, the relative EEG signal power for the left *(W_Lmn_)* and the right (*W_Rmn_*) hemispheric symmetric channels (indexed as *L* and *R*) were calculated for each EEG frequency band as shown in Equations 1–3 (30).


(3) $$W_{Lmn}^{'}=\sum_{f=f_{1}}^{f_{2}}\;S_{Lmn}\;/\;\sum_{f=0.5Hz}^{45Hz}S_{Lmn}$$

(4) $$W_{Rmn}^{'}=\sum_{f=f_{1}}^{f_{2}}\;S_{Rmn}\;/\;\sum_{f=0.5Hz}^{45Hz}S_{Rmn}$$

(5) $$A_{mn}\left(f_{1},f_{2}\right)=\;\frac{W_{Lmn}^{'}-W_{Rmn}^{'}}{W_{Lmn}^{'}+W_{Rmn}^{'}}x100$$

Higher asymmetry scores indicate greater relative activation of the left hemisphere. The asymmetry analysis was conducted on symmetric EEG channel pairs: Fp1-Fp2, F7-F8, F3-F4, C3-C4, T3-T4, T5-T6, P3-P4, and O1–O2.

### 2.4 Statistical analysis

All statistical analyses were performed using SPSS statistical software, version 22.0 (SPSS Institute, Inc., Chicago, IL, USA). Absolute power values, relative power values, and interhemispheric asymmetry values were analyzed. Results were evaluated for each channel and frequency band to compare the depression and healthy control groups. Depending on the dataset distribution, either an independent sample t-test or Mann-Whitney U test was applied. MATLAB (2023b) and its related toolboxes were used for all data analyses and visualizations. Data analysis, visualization, and design were performed in this manuscript did not make use of any generative AI technologies.

## 3 Results

### 3.1 Absolute power analysis results

**Figure 3:**
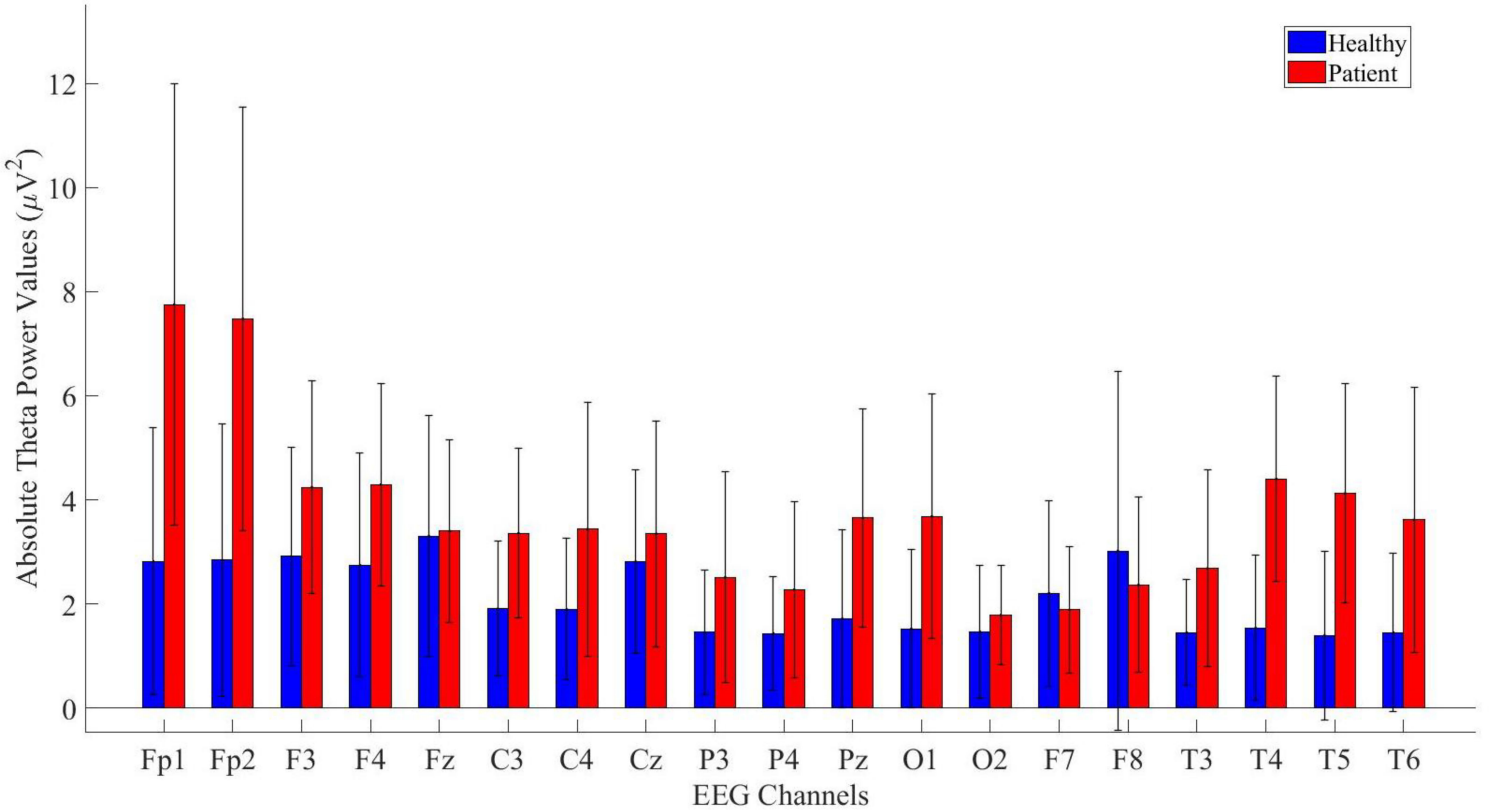
Comparative analysis of absolute theta power values showing statistically significant differences between patients and healthy volunteers.

In the study, absolute power values of theta frequency band signals were computed for all electrodes. The analysis was conducted for both healthy and patients. The results of the analysis were then compared and are presented in **Figure 3**.

The power values of the patient and healthy groups were statistically analyzed. The results indicated that patients exhibited higher power values in the Fp1, Fp2, F3, F4, Fz, C3, C4, Cz, P3, P4, Pz, O1, T3, T4, T5, T6 and T6 channels. The detailed statistical analysis results are provided in **Table 2**. 

**Table 2:** Statistical analysis results of theta absolute power values of patient and healthy volunteers.

Channel	Patients	Control.	p value
Fp1	7.752 ± 4.240	2.818 ± 2.566	**<.00001**
Fp2	7.477 ± 4.070	2.841 ± 2.619	**<.00001**
F3	4.244 ± 2.047	2.913 ± 2.088	**<.00001**
F4	4.295 ± 1.943	2.746 ± 2.153	**<.00001**
Fz	3.401 ± 1.757	3.305 ± 2.311	**<.00001**
C3	3.362 ± 1.622	1.910 ± 1.297	**<.00001**
C4	3.437 ± 2.438	1.898 ± 1.359	**<.00001**
Cz	3.349 ± 2.172	2.815 ± 1.763	**<.00001**
P3	2.514 ± 2.020	1.457 ± 1.199	**<.00001**
P4	2.278 ± 1.692	1.436 ± 1.096	**<.00001**
Pz	3.653 ± 2.093	1.719 ± 1.706	**<.00001**
O1	3.685 ± 2.353	1.525 ± 1.519	**<.00001**
O2	1.788 ± 0.954	1.467 ± 1.279	NS
F7	1.887 ± 1.213	2.209 ± 1.780	NS
F8	2.370 ± 1.678	3.020 ± 3.452	NS
T3	2.685 ± 1.885	1.452 ± 1.012	**<.001**
T4	4.403 ± 1.972	1.540 ± 1.395	**<.00001**
T5	4.126 ± 2.109	1.399 ± 1.621	**<.00001**
T6	3.618 ± 2.542	1.454 ± 1.515	**<.00001**

“NS” indicates non-significant results (p ≥ 0.05). Bold values in the tables indicate statistically significant results (*p* < 0.05).

**Figure 4:**
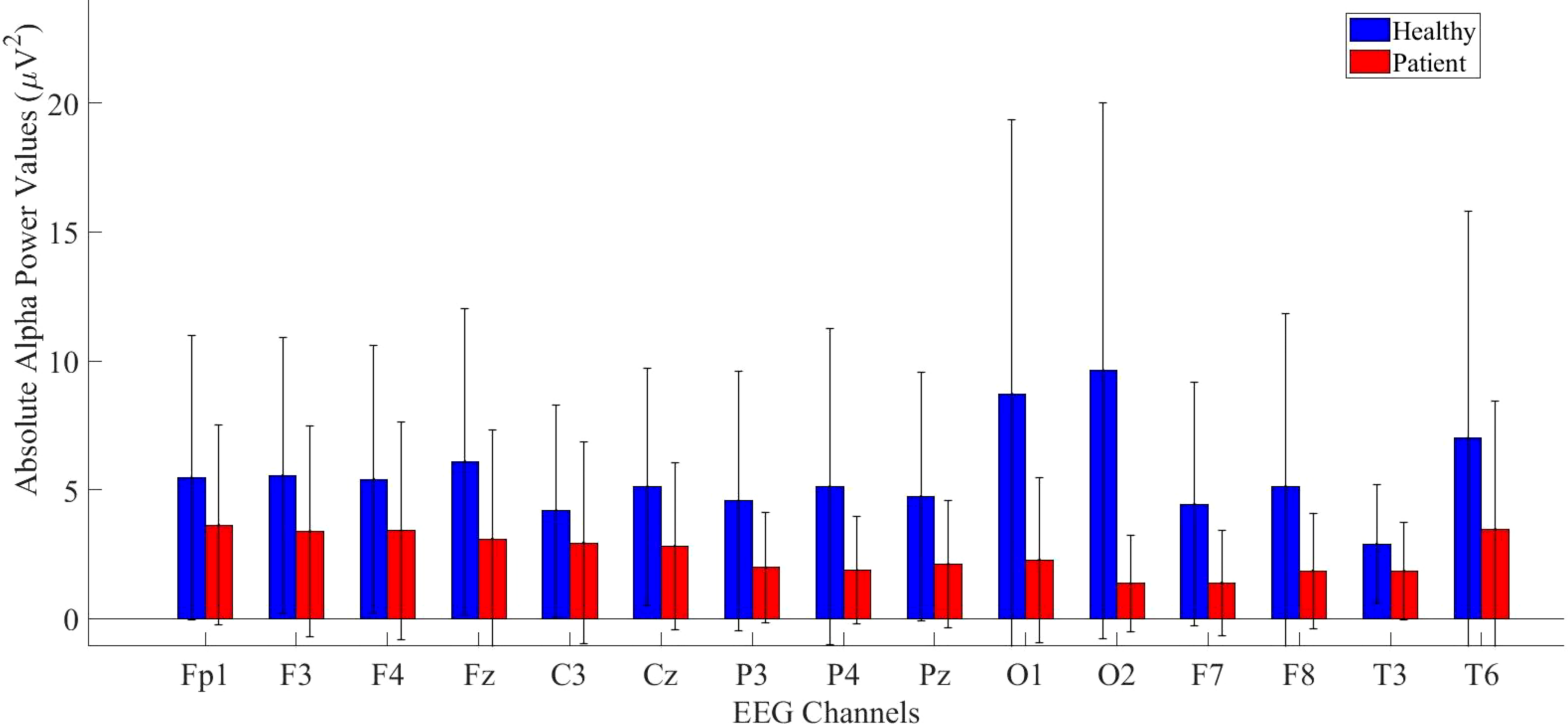
Comparative analysis of absolute alpha power values showing statistically significant differences between patients and healthy volunteers.

Same analysis were performed also for alpha power values and the results are presented in **Figure 4**.

According to statistical analysis, a significantly higher difference was found in the healthy group. The detailed results are provided in **Table 3**.

**Table 3:** Statistical analysis results of absolute alpha power values of patient and healthy volunteers.

Channel	Patients	Control	p value
Fp1	3.626 ± 3.868	5.462 ± 5.515	**<.001**
Fp2	3.807 ± 4.311	7.719 ± 21.348	NS
F3	3.386 ± 4.084	5.549 ± 5.339	**<.001**
F4	3.411 ± 4.234	5.408 ± 5.174	**<.001**
Fz	3.095 ± 4.213	6.088 ± 5.921	**<.001**
C3	2.943 ± 3.898	4.190 ± 4.101	**<.001**
C4	3.034 ± 3.985	3.933 ± 3.707	NS
Cz	2.813 ± 3.235	5.118 ± 4.578	**<.00001**
P3	1.980 ± 2.136	4.570 ± 5.032	**<.00001**
P4	1.874 ± 2.087	5.121 ± 6.149	**<.00001**
Pz	2.111 ± 2.453	4.736 ± 4.814	**<.00001**
O1	2.279 ± 3.200	8.710 ± 10.635	**<.00001**
O2	1.364 ± 1.887	9.621 ± 10.391	**<.00001**
F7	1.386 ± 2.033	4.431 ± 4.724	**<.00001**
F8	1.857 ± 2.232	5.126 ± 6.724	**<.0001**
T3	1.851 ± 1.901	2.894 ± 2.298	**<.001**
T4	3.970 ± 4.699	2.599 ± 1.934	NS
T5	3.587 ± 4.447	5.095 ± 6.483	NS
T6	3.464 ± 4.978	7.000 ± 8.793	**<.001**

“NS” indicates non-significant results (p ≥ 0.05). Bold values in the tables indicate statistically significant results (*p* < 0.05).

**Figure 5:**
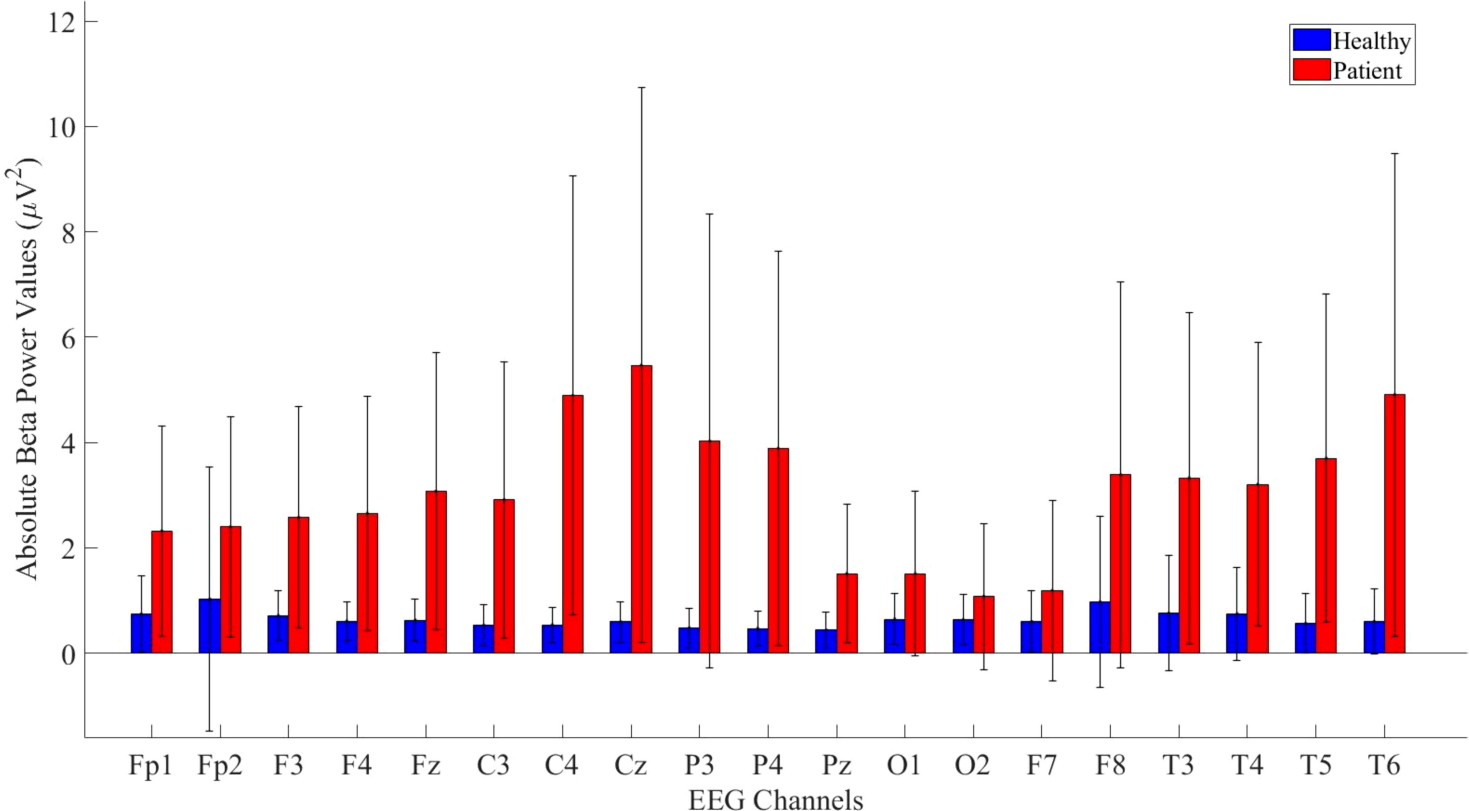
Comparative analysis of absolute beta power values showing statistically significant differences between patients and healthy volunteers.

Beta band values were also included in the study due to significant differences found in the Fp1, F3, F4, Fz, C3, Cz, P3, P4, Pz, O1, O2, F7, F8, T3, and T6 channels. The patient group exhibited higher absolute power values across most bands, as illustrated in **Figure 5**.

The statistical analysis results of absolute beta power values are provided in **Table 4**.

**Table 4:** Statistical analysis results of absolute beta power values of patient and healthy volunteers.

Channel	Patients	Control.	p value
FP1	2.322 ± 1.996	0.745 ± 0.730	**<.00001**
FP2	2.397 ± 2.094	1.027 ± 2.500	**<.00001**
F3	2.581 ± 2.099	0.716 ± 0.468	**<.00001**
F4	2.657 ± 2.226	0.609 ± 0.367	**<.00001**
FZ	3.075 ± 2.630	0.629 ± 0.392	**<.00001**
C3	2.912 ± 2.617	0.534 ± 0.388	**<.00001**
C4	4.894 ± 4.164	0.538 ± 0.337	**<.00001**
CZ	5.465 ± 5.268	0.598 ± 0.383	**<.00001**
P3	4.027 ± 4.309	0.483 ± 0.377	**<.00001**
P4	3.889 ± 3.745	0.471 ± 0.321	**<.00001**
PZ	1.509 ± 1.315	0.442 ± 0.337	**<.00001**
O1	1.510 ± 1.564	0.647 ± 0.479	**<.00001**
O2	1,.078 ± 1.384	0.636 ± 0.480	**<.00001**
F7	1.189 ± 1.716	0.603 ± 0.580	**<.00001**
F8	3.385 ± 3.662	0.972 ± 1.621	**<.00001**
T3	3.329 ± 3.143	0.759 ± 1.091	**<.00001**
T4	3.204 ± 2.689	0.749 ± 0.885	**<.00001**
T5	3.700 ± 3.116	0.564 ± 0.566	**<.00001**
T6	4.904 ± 4.583	0.604 ± 0.622	**<.00001**

Bold values in the tables indicate statistically significant results (*p* < 0.05).

### 3.2 Relative power analysis results

**Figure 6:**
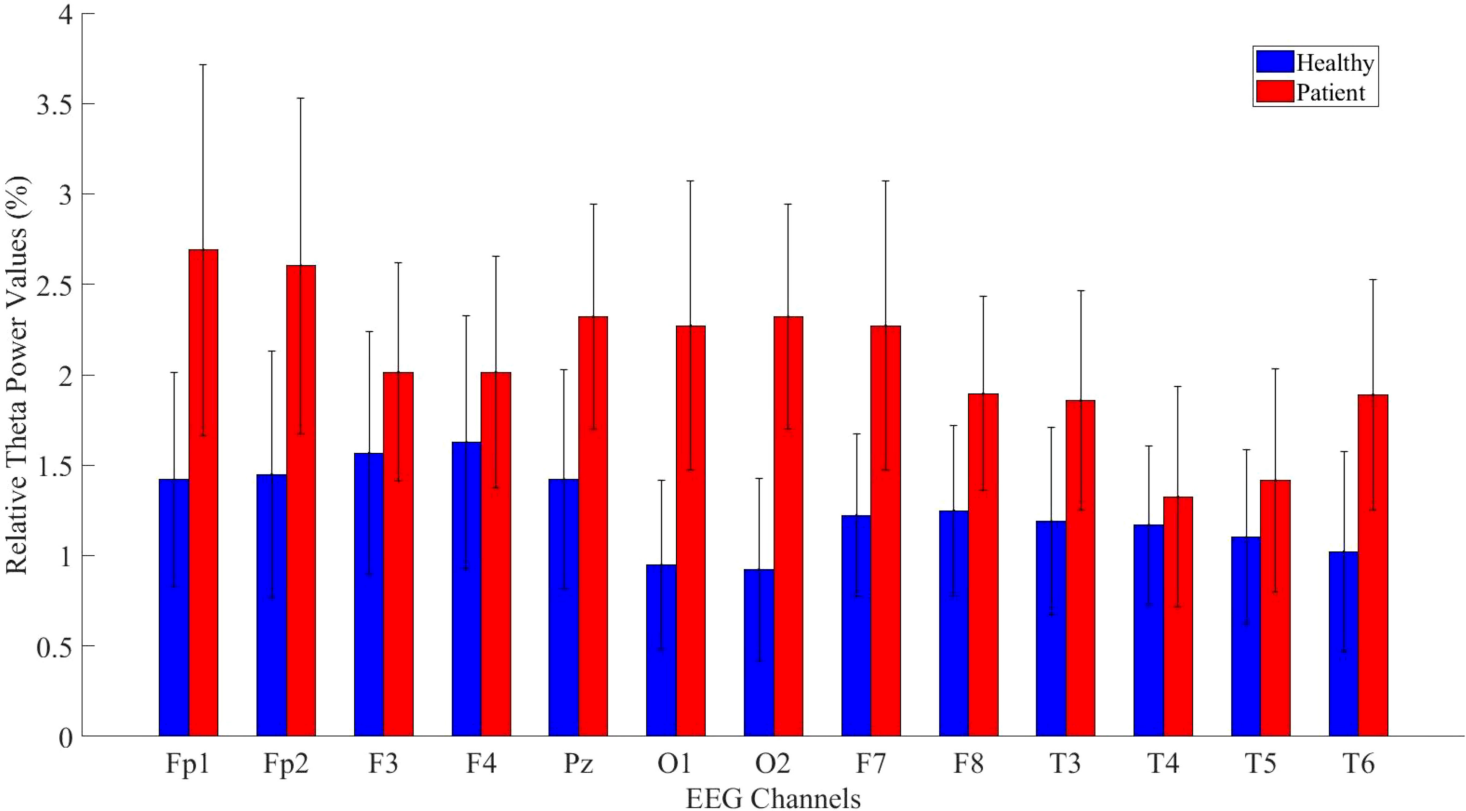
Comparative analysis of relative theta power values showing statistically significant differences between patients and healthy volunteers.

After completing the analyses of absolute power values, relative power analyses—another crucial parameter in neurophysiological research—were calculated for the theta, alpha, and beta bands. A significant difference was found across all channels for the theta band, as shown in **Figure 6**.

The statistical analysis results for the theta band are presented in **Table 5**. The results indicate a significant difference between patient and healthy groups across most channels.

**Table 5:** Statistical analysis results of relative theta power values of patient and healthy volunteers.

Channel	Patients	Control	p value
Fp1	2.691 ± 1.026	1.420 ± 0.5919	**< 0.00001**
Fp2	2.603 ± 0.927	1.449 ± 0.681	**< 0.00001**
F3	2.015 ± 0.605	1.568 ± 0.671	**< 0.00001**
F4	2.015 ± 0.641	1.627 ± 0.699	**< 0.00001**
FZ	1.680 ± 0.613	1.879 ± 0.747	NS
C3	1.713 ± 0.615	1.542 ± 0.576	NS
C4	1.272 ± 0.653	1.536 ± 0.595	NS
Cz	1.251 ± 0.619	1.878 ± 0.689	NS
P3	1.191 ± 0.566	1.296 ± 0.536	NS
P4	1.180 ± 0.488	1.260 ± 0.605	NS
Pz	2.321 ± 0.623	1.422 ± 0.607	**<.00001**
O1	2.272 ± 0.798	0.948 ± 0.468	**<.00001**
O2	2.321 ± 0.623	0.923 ± 0.506	**<.0001**
F7	2.272 ± 0.798	1.223 ± 0.449	**<.00001**
F8	1.896 ± 0.538	1.249 ± 0.469	**<.00001**
T3	1.858 ± 0.608	1.189 ± 0.520	**<.00001**
T4	1.325 ± 0.610	1.169 ± 0.440	**<.00001**
T5	1.416 ± 0.618	1.101 ± 0.483	**<.00001**
T6	1.890 ± 0.637	1.022 ± 0.552	**<.00001**

“NS” indicates non-significant results (p ≥ 0.05). Bold values in the tables indicate statistically significant results (*p* < 0.05).

**Figure 7:**
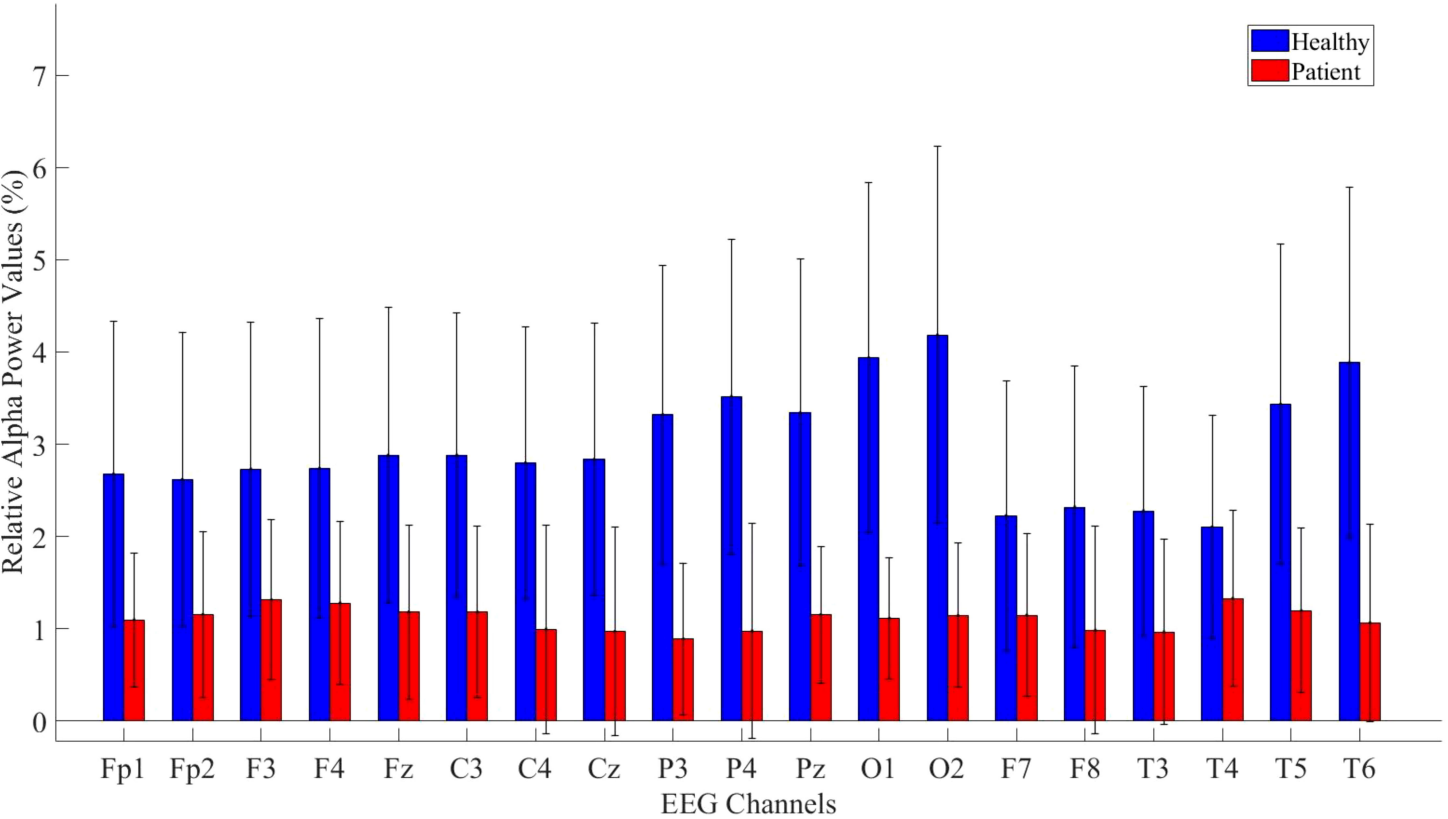
Comparative analysis of relative alpha power values showing statistically significant differences between patients and healthy volunteers.

Relative alpha power values were computed for both the patient and healthy groups. The power values in the alpha frequency band were higher in MDD patients compared to healthy controls. The analysis results are illustrated in **Figure 7**.

The statistical analysis revealed a significant difference between the groups, with detailed results provided in **Table 6**.

**Table 6:** Statistical analysis results of relative alpha power values of patient and healthy volunteers.

Channel	Patients	Control	p value
Fp1	1.097 ± 0.727	2.677 ± 1.658	**<.00001**
Fp2	1.155 ± 0.895	2.617 ± 1.595	**<.00001**
F3	1.315 ± 0.870	2.730 ± 1.596	**<.00001**
F4	1.279 ± 0.879	2.739 ± 1.627	**<.00001**
FZ	1.181 ± 0.945	2.880 ± 1.601	**<.00001**
C3	1.183 ± 0.926	2.882 ± 1.541	**<.00001**
C4	0.996 ± 1.131	2.798 ± 1.469	**<.00001**
Cz	0.971 ± 1.133	2.834 ± 1.479	**<.00001**
P3	0.891 ± 0.822	3.321 ± 1.619	**<.00001**
P4	0.976 ± 1.163	3.517 ± 1.704	**<.00001**
Pz	1.152 ± 0.742	3.340 ± 1.665	**<.00001**
O1	1.115 ± 0.656	3.937 ± 1.898	**<.00001**
O2	1.145 ± 0.782	4.183 ± 2.043	**<.00001**
F7	1.148 ± 0.886	2.225 ± 1.462	**<.00001**
F8	0.984 ± 1.126	2.319 ± 1.530	**<.00001**
T3	0.965 ± 1.005	2.278 ± 1.352	**<.00001**
T4	1.330 ± 0.949	2.103 ± 1.213	**<.00001**
T5	1.196 ± 0.894	3.432 ± 1.738	**<.00001**
T6	1.065 ± 1.067	3.883 ± 1.897	**<.00001**

Bold values in the tables indicate statistically significant results (*p* < 0.05).

**Figure 8:**
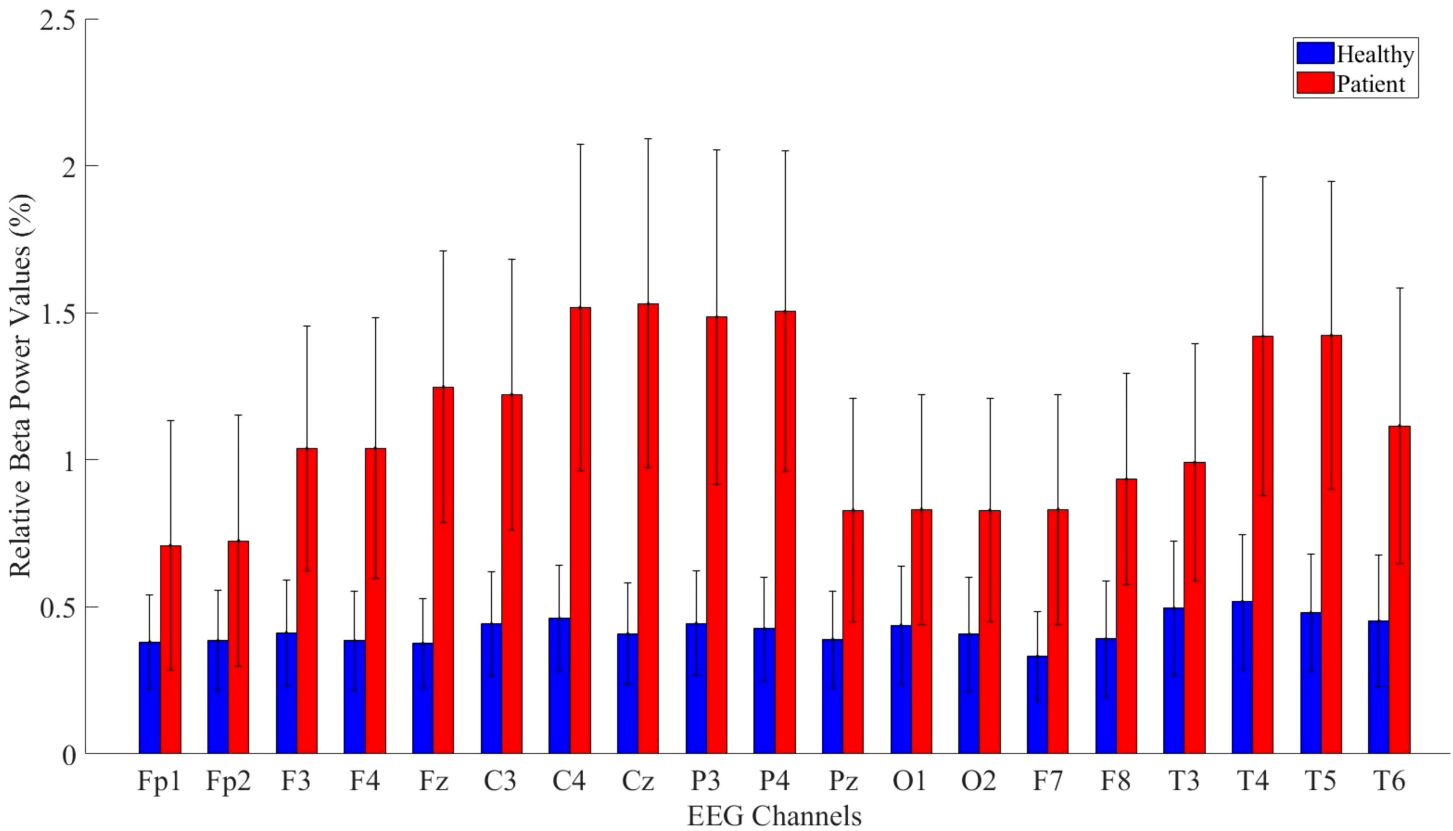
Comparative analysis of relative beta power values showing statistically significant differences between patients and healthy volunteers.

Relative beta power values were computed for both the patient and healthy groups. The power values in the beta frequency band were higher in MDD patients compared to healthy controls. The analysis results are presented in **Figure 8**.

Detailed statistical analysis results comparing the beta band values between the patient and healthy groups are provided in **Table 7**.

**Table 7:** Statistical analysis results of relative beta power values of patient and healthy volunteers.

Channel	Patients	Control	p value
Fp1	0.709 ± 0.425	0.381 ± 0.159	**<.00001**
Fp2	0.725 ± 0.428	0.386 ± 0.171	**<.00001**
F3	1.039 ± 0.415	0.412 ± 0.179	**<.00001**
F4	1.040 ± 0.442	0.385 ± 0.168	**<.00001**
FZ	1.248 ± 0.462	0.376 ± 0.151	**<.00001**
C3	1.222 ± 0.461	0.442 ± 0.176	**<.00001**
C4	1.518 ± 0.555	0.461 ± 0.179	**<.00001**
Cz	1.531 ± 0.560	0.409 ± 0.172	**<.00001**
P3	1.485 ± 0.570	0.444 ± 0.178	**<.00001**
P4	1.505 ± 0.545	0.426 ± 0.175	**<.00001**
Pz	0.829 ± 0.379	0.390 ± 0.164	**<.00001**
O1	0.832 ± 0.391	0.438 ± 0.200	**<.00001**
O2	0.829 ± 0.379	0.407 ± 0.195	**<.00001**
F7	0.832 ± 0.391	0.332 ± 0.152	**<.00001**
F8	0.934 ± 0.360	0.391 ± 0.197	**<.00001**
T3	0.991 ± 0.404	0.495 ± 0.229	**<.00001**
T4	1.420 ± 0.541	0.518 ± 0.228	**<.00001**
T5	1.424 ± 0.523	0.480 ± 0.199	**<.00001**
T6	1.116 ± 0.469	0.452 ± 0.223	**<.00001**

Bold values in the tables indicate statistically significant results (*p* < 0.05).

### 3.3 Asymmetry analysis results

Interhemispheric asymmetry values provide crucial information about an individual’s mental state and can serve as biomarkers. While alpha asymmetry is commonly used in the literature, we also computed theta and beta asymmetry values. Significant differences were found across the theta, alpha, and beta bands. The analysis results are presented in **Tables 8**–**10**.

**Table 8:** Statistical analysis results of theta asymmetry values of patients.

Channel	Patients	Control	p value
Fp1-Fp2	1.442 ± 5.688	0.355 ± 10.385	NS
F7-F8	7.856 ± 12.077	1.555 ± 15.286	**<.00001**
F3-F4	0.494 ± 5.970	1.157 ± 17.183	NS
C3-C4	16.460 ± 14.140	0.440 ± 7.593	**<.00001**
T3-T4	17.754 ± 18.006	0.784 ± 16.808	**<.00001**
T5-T6	12.862 ± 24.491	5.727 ± 16.509	**<.00001**
P3-P4	-0.710 ± 13.522	2.717 ± 8.877	NS
O1-O2	-2.420 ± 10.832	3.034 ± 14.757	NS

“NS” indicates non-significant results (p ≥ 0.05). Bold values in the tables indicate statistically significant results (*p* < 0.05).

**Table 9:** Statistical analysis results of alpha asymmetry values of patients.

Channel	Patients	Control	p value
Fp1-Fp2	-0.607 ± 6.261	0.802 ± 14.729	NS
F7-F8	13.919 ± 18.757	0.512 ± 17.335	**<.00001**
F3-F4	1.707 ± 3.467	1.376 ± 18.166	NS
C3-C4	15.425 ± 13.306	1.275 ± 9.873	**<.00001**
T3-T4	20.987 ± 18.724	2.890 ± 18.024	**<.00001**
T5-T6	10.552 ± 20.933	5.836 ± 16.305	**<.00001**
P3-P4	0.680 ± 12.134	2.885 ± 9.765	NS
O1-O2	-0.642 ± 10.205	2.864 ± 12.262	**<.05**

“NS” indicates non-significant results (p ≥ 0.05). Bold values in the tables indicate statistically significant results (*p* < 0.05).

**Table 10:** Statistical analysis results of beta asymmetry values of patients.

Channel	Patients	Control	p value
Fp1-Fp2	-1.234 ± 10.300	-0.391 ± 14.248	NS
F7-F8	-7.752 ± 12.737	-6.527 ± 17.770	NS
F3-F4	0.108 ± 4.928	2.667 ± 19.700	NS
C3-C4	10.110 ± 9.364	-2.275 ± 8.735	**<.00001**
T3-T4	16.736 ± 17.949	-2.025 ± 19.439	**<.00001**
T5-T6	13.741 ± 12.557	5.007 ± 15.882	**<.005**
P3-P4	-2.056 ± 6.831	2.221 ± 6.764	**<.005**
O1-O2	0.214 ± 6.764	3.726 ± 6.764	NS

“NS” indicates non-significant results (p ≥ 0.05). Bold values in the tables indicate statistically significant results (*p* < 0.05).

## 4 Discussion

The main objective of this study was to explore the impact of Major Depressive Disorder (MDD) on brain power spectral density. To achieve this, we computed absolute and relative power spectral values using the Welch method. The results revealed an increase in absolute power in all channels except O2, F7, and F8. For relative power, an increase was observed in all channels except C4, Cz, P3, and P4. Analyses were conducted for the theta, alpha, and beta frequency bands, as these regions provide crucial insights into MDD. In the beta band, a statistically significant power increase was observed across all channels in the patient group. Conversely, for the alpha band, a statistically significant decrease in power was detected in almost all channels for MDD patients.

The analysis of the theta, alpha, and beta frequency bands offers vital neuroanatomical and neurophysiological information about the central nervous system. Several studies have demonstrated a relationship between theta and alpha waves and memory function. For example, Buzsaki, Jansen, and Lisman et al. detected theta oscillations during information encoding and spatial navigation (39, 40). Additionally, neurophysiological research suggests that theta waves are generated in the amygdala and hippocampal CA1 region during emotional events (41, 42). Higher theta band asymmetry in frontal and temporal regions may indicate disruptions in interhemispheric networks. Theta activity is closely associated with connectivity, memory processing, attention, and emotional regulation. Therefore, the theta asymmetry values observed in this research may reflect the impact of MDD on these critical brain functions. Neuroimaging studies in individuals with MDD have shown reductions in hippocampal volume and dysfunctions in the orbitofrontal cortex, anterior cingulate cortex (43), dorsolateral prefrontal cortex (44), amygdala (45). Furthermore, these studies indicate that MDD impairs cognitive functions such as memory (46), executive functioning and attention (47). The beta band provides important insights into neural mechanisms, including cortical activation and deficits in sustaining attentional processes (48) Additionally, abnormal beta band power has been associated with heightened arousal levels (49).

Alpha waves have also been linked to memory processes and cortical activity. Specifically, higher alpha activity is associated with enhanced memory performance (50, 51). There is strong evidence that alpha power increases during working memory tasks (52) and attention processes (53). As EEG power is a biomarker indicating the activity and performance of cortical processing, it is significantly correlated with cognitive and memory performance. Strong alpha activity generally reflects good memory performance, while lower theta power may indicate poor memory performance (50). Previous researches reported that frontal asymmetry can be used as a risk index for emotion related disorder including MDD (54). Alpha asymmetry values were associated with emotional regulations and depressive symptom severity in individuals (55). Beta asymmetry values were also used as a biomarker of MDD and especially left hemispheric hyper activity was found in MDD (56).

Our findings align with these observations and add robust statistical evidence to the literature. When considering neuroimaging studies on the effects of MDD and EEG band power investigations together, our results provide strong support for existing research.

A previous study investigating treatment effectiveness in deep brain stimulation surgery employed the FFT method. In contrast, the Welch method, as used in our study, segments data rather than analyzing the entire dataset at once, providing smoother results. Additionally, we computed asymmetry values for both lower and upper frequency bands, comparing them between healthy individuals and patients. While alpha and, to some extent, theta asymmetry values are commonly used as biomarkers in the literature, our study also found significant results for beta asymmetry values.

Knott et al. computed absolute, relative power, and asymmetry values using the conventional FFT method and identified differences in relative beta power and alpha power asymmetry indices (57). However, they did not report significant findings for other frequency bands. Tas et al. examined differences in power, cordance, and coherence between unipolar and bipolar depression but did not find any significant differences in power values (58). Roh et al., investigating attentional impairments in MDD patients using the FFT method, found a negative correlation between inattention scores and power values (59).

This study employed the Welch method to investigate MDD and its impact on EEG power spectra. As an advanced version of the classical FFT method, the Welch approach provides smoother power estimates. To our knowledge, this is the first study to apply the Welch method in MDD research. Both absolute and relative power analyses were conducted separately and our findings corroborate neuroimaging studies that report hippocampal volume reduction and memory and attentional disorders in MDD. Additionally, our results show that MDD is associated with dysfunction in the orbitofrontal and dorsolateral prefrontal cortices, as well as the amygdala. Furthermore, significant differences in theta, alpha, and beta asymmetry values between patients and healthy controls were observed, suggesting that these asymmetry values may serve as potential biomarkers for the early diagnosis of depression.

Disruptions in the default mode network (DMN), which is linked to self-referential cognition and emotional regulation, may be the cause of abnormal theta and alpha power in MDD patients. Prior research has shown that ruminative thought processes and cognitive deficits in MD are linked to DMN connectivity (60). In addition to this functional connectivity measure in DMN was found positively related to MDD severity (61). Abnormalities in emotional regulation are a key characteristic of MDD (62). Several studies in the literature have explored the relationship between theta and alpha bands and emotional processes. It has been suggested that altered theta synchronization may be associated with disruptions in emotional regulation (63).

It is crucial to understand that additional research is necessary to evaluate the possible marker’s usefulness at the individual or case level, even if this study focuses on statistical significance at the group level. In order to guarantee the marker’s applicability in clinical or real-world settings, future research will attempt to investigate case-specific analyses, such as sensitivity, specificity, and effect sizes. This will lessen the possibility that, when used outside of group comparisons, the marker’s significance may be overestimated. While this study focused on comparing EEG power and asymmetry values between patients with MDD and healthy controls, future research will aim to explore how demographic and clinical factors, effect EEG results. For example, understanding the relationship between symptom severity and EEG power values may provide deeper insights into the neurophysiological mechanisms underlying MDD. Additionally, effects of gender differences on EEG power asymmetry, which have been noted in some prior studies, warrant further investigation to clarify their role in MDD.

## Data Availability

The original contributions presented in the study are included in the article/supplementary material. Further inquiries can be directed to the corresponding author.
